# The Impact of Early Deafness on Brain Plasticity: A Systematic Review of the White and Gray Matter Changes

**DOI:** 10.3389/fnins.2020.00206

**Published:** 2020-03-30

**Authors:** Marie Simon, Emma Campbell, François Genest, Michèle W. MacLean, François Champoux, Franco Lepore

**Affiliations:** ^1^Département de Psychologie, Centre de Recherche en Neuropsychologie et Cognition, Université de Montréal, Montreal, QC, Canada; ^2^École d'Orthophonie et d'Audiologie, Université de Montréal, Montreal, QC, Canada

**Keywords:** deafness, brain development, neuroplasticity, neuroimaging, language acquisition

## Abstract

**Background:** Auditory deprivation alters cortical and subcortical brain regions, primarily linked to auditory and language processing, resulting in behavioral consequences. Neuroimaging studies have reported various degrees of structural changes, yet multiple variables in deafness profiles need to be considered for proper interpretation of results. To date, many inconsistencies are reported in the gray and white matter alterations following early profound deafness. The purpose of this study was to provide the first systematic review synthesizing gray and white matter changes in deaf individuals.

**Methods:** We conducted a systematic review according to the Preferred Reporting Items for Systematic Reviews and Meta-Analyses (PRISMA) statement in 27 studies comprising 626 deaf individuals.

**Results:** Evidence shows that auditory deprivation significantly alters the white matter across the primary and secondary auditory cortices. The most consistent alteration across studies was in the bilateral superior temporal gyri. Furthermore, reductions in the fractional anisotropy of white matter fibers comprising in inferior fronto-occipital fasciculus, the superior longitudinal fasciculus, and the subcortical auditory pathway are reported. The reviewed studies also suggest that gray and white matter integrity is sensitive to early sign language acquisition, attenuating the effect of auditory deprivation on neurocognitive development.

**Conclusions:** These findings suggest that understanding cortical reorganization through gray and white matter changes in auditory and non-auditory areas is an important factor in the development of auditory rehabilitation strategies in the deaf population.

## Introduction

Neuroplasticity is an intrinsic property of the brain (Dennis et al., 2013) and refers to the brain's ability to reorganize itself in response to learning and the environmental interactions throughout life (Pascual-Leone et al., 2005). Early neuroplasticity increases the vulnerability of the immature brain, possibly leading to adverse development (Dennis et al., 2014). Thus, neuroplasticity can also be associated with a neurodevelopmental and behavioral pathology (Gilmore et al., 2018), involving both functional and structural modifications, and can lead to behavioral consequences (May, 2011). Therefore, given the absence of experience in the auditory cortex of congenitally deaf children, early deafness constitutes an excellent model to study neuroplasticity mechanisms in the human brain.

In neurotypical children, ontogenetic events support the development of the brain through neurogenesis, axonal and dendritic growth, synaptogenesis, synaptic pruning, and myelination (Anderson et al., 2014). These events are highly interdependent, such that perturbation in one specific area of development can have long-term effects on the brain's structural and functional integrity (Grantham-McGregor et al., 2007). Indeed, intrauterine and early childhood developments are critical to the proper maturation of cognitive abilities and behaviors, as brain development is characterized mainly by reorganization, “fine-tuning,” or remodeling of primary circuits and networks after the age of two (Gilmore et al., 2018). Brain regions associated with primary functions such as perception (e.g., vision and audition) and gross motor abilities mature first and are followed by areas supporting spatial orientation and language development; brain areas involved in executive function, attention, and motor coordination appear to mature last (Gogtay et al., 2004; Grantham-McGregor et al., 2007).

Several studies have demonstrated a developmental decrease of synaptic plasticity in the auditory cortex after early deafness (for a review, see Kral and Sharma, 2012). Consequently, neuroplastic changes occur at the youngest age in early deaf children and are generally related to a sensitive period (Sharma et al., 2005). In the particular context of early deprivation, this sensitive period corresponds to a window during which experience is critical for the development of sensory functions (Kral, 2013). In deaf children, this sensitive period mainly occurs up to the third year of life and corresponds to a critical limit for auditory rehabilitation, especially as it relates to cochlear implantation (Kral, 2013). Based on these lines of evidence, the consequences of auditory deprivation on cortical maturation in congenitally or prelingually deaf children is of high importance for auditory rehabilitation, particularly for language acquisition and neurocognitive development.

With magnetic resonance imaging (MRI), numerous studies have acquired *in vivo* data to describe a plethora of cerebral structures in deaf individuals in comparison with hearing peers. *Morphometric analysis* was one of the first techniques used to describe anatomical reorganization in deaf individuals (Emmorey et al., 2003; Penhune et al., 2003). This technique allows the classification of cerebral tissues whereby the gray matter, white matter, and cerebrospinal fluid volumes can be calculated (Filipek et al., 1994). Subsequently, *voxel-based morphometry* (VBM) was developed to allow voxel-by-voxel assessment of tissue density in the white and gray matter in typical and atypical brains (Wright et al., 1995). Complementary to VBM is *cortical thickness* (CT), which measures the distance between the white and gray matter and the distance between the gray matter and the dura mater (He et al., 2007), and *tensor-based morphometry* (TBM), which enables measurement of volume differences in the brain (Ashburner and Friston, 2001). *Diffusion MRI* is used to analyze the integrity of the white matter structures by estimating fiber structure through water molecule diffusion (e.g., Mori and Zhang, 2006; Mukherjee et al., 2008). For example, *diffusion tensor imaging* (DTI) determines whether or not water molecules diffuse in all directions and specifies the preferred diffusion direction within a given tract. The general index of the structural integrity and directionality of axonal fibers within a voxel [fractional anisotropy (FA)] is the most frequently reported DTI measure. Finally, as an alternative to DTI, *diffusion kurtosis imaging* (DKI) allows the measure of Gaussian and, more particularly, non-Gaussian properties of water diffusion (Lu et al., 2006).

In this systematic review, we first report the current state of knowledge regarding gray and white matter changes found in deaf individuals through various neuroimaging techniques (volumetry, VBM, TBM, CT, DTI, and DKI). We then describe these structural changes as they relate to factors known to influence the extent of cortical plasticity. Finally, we interpret the reported findings in the context of recent advances and present our current understanding of these macroscopic cortical plasticity phenomena. We also discuss the predictive value of structural changes relating to language acquisition and neurocognitive development in deaf individuals, as well as how it can guide rehabilitation strategies.

## Methods

This systematic review was conducted according to the Preferred Reporting Items for Systematic Reviews and Meta-Analyses (PRISMA) statement (Moher et al., 2009).

### Inclusion Criteria

Studies were eligible if they included (1) a structural, anatomical, or morphometric MRI brain analysis technique; (2) congenitally or prelingually deaf adults, adolescents, or children; and (3) participants presenting severe to profound bilateral hearing loss. Studies involving unilateral or late-acquired deafness as well as animal data were excluded. Only articles published in a peer-reviewed journal in English or translated into English were considered.

### Search Strategy

Online searches on PubMed, including PubMed Central and Medline, PsycNET, including PsycINFO and PsycARTICLES, and Web of Science (Core Collection) were performed in April 2017, repeated in September 2017 and in April 2018 with relevant search terms. Search terms were: (“Deaf” OR “Hearing loss”) AND (“Voxel-based morphometry” OR “VBM” OR “Diffusion tensor imaging” OR “DTI” OR “Cortical thickness” OR “White matter” OR “Gray matter” OR “Morphometric” OR “Neuroimaging”). All database literature coverage ranged from 1974 to present, and no automatic filter was used for publication type (journal article, case report, conference findings, review, etc.).

### Study Selection

**Figure 1:**
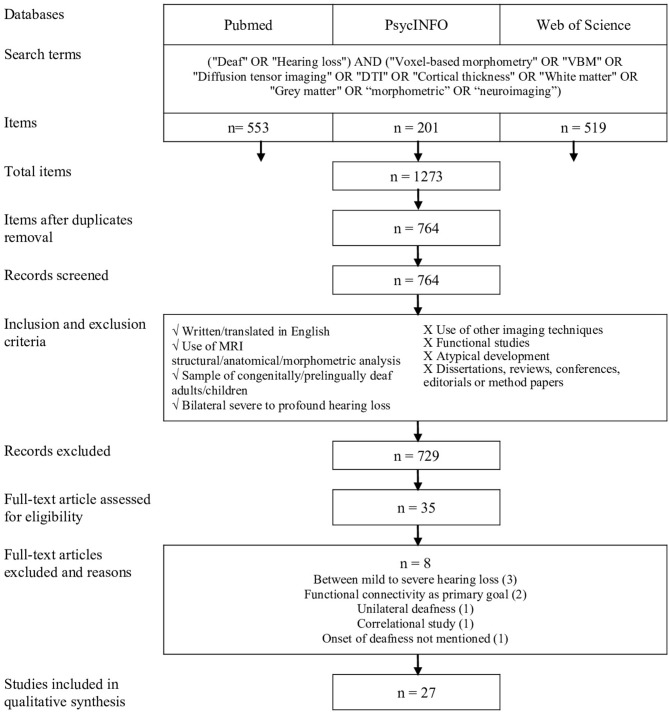
Procedure for systematic review inspired by the PRISMA protocol (Moher et al., 2009).

The study selection procedure is presented in Figure 1. All studies were compiled to ensure the removal of duplicates; two distinct reviewers verified this procedure. Then, the first reviewer selected potential studies on the basis of title, abstract, and publication type. The second reviewer verified the previous selection and all articles that had been considered incompatible. After this screening, the two reviewers evaluated all the articles for full-text eligibility.

### Data Collection Process

Extracted data for each study included (1) meta-study information (e.g., name of the authors and year of publication); (2) sample characteristics, including demographics (e.g., age) and hearing loss variables (e.g., onset and type of hearing loss, and communication preference); (3) neuroimaging analysis (e.g., DTI, CT, and VBM), measure used [e.g., region of interest (ROI) or whole brain], MRI scanner strength, and coordinate reference system; and (4) method and results of any significant (at least *p* < 0.05) group-based comparisons in the neuroimaging measures. For each brain region, reviewers took note of whether a significant alteration was found regarding volume, CT, FA (the common index of structural integrity and directionality of axonal fibers within a voxel), axial diffusivity (AD; reflecting integrity of microtubules along axon), radial diffusivity (RD; indexing levels of myelination), mean diffusivity (MD; reflecting mathematical combination of the RD and AD), or mean kurtosis (MK; reflecting structural changes in both anisotropic and isotropic tissue). Reviewers also specified if changes occurred in the white or gray matter and in a specific hemisphere. Owing to the lack of specific coordinates in most studies, Montreal Neurological Institute (MNI) or Talairach coordinates were not compiled, and only brain regions were considered. Pertinent details from each study are presented in Table 1.

**Table 1:** Main characteristics of selected articles for systematic review.

**References**	**Participants**							**MNI scanner strength (Testla)**	**Brain imaging technics**	**Analysis**	**Reference**	**Statistical correction**
	**Deaf (N)**	**NH (N)**	**Age (SD) (years)**	**Hearing loss**	**Onset**	**Hearing Aids (HA)**	**Communication preference**					
Allen et al. (2008)	25	25 16 CODA	28.3 (4.5)	Profound	Congenital	HA> 2	NSL	1.5	MVA	ROI	NM	Bonferroni
Allen et al. (2013)	25	25 16 CODA	28.3 (4.5)	Profound	Congenital	HA > 2	NSL	1.5	MVA	ROI	NM	Bonferroni
Amaral et al. (2016)	15	16	20.4 (NM)	Profound	Congenital	No HA	NSL	3	VA	ROI	NM	Greenhouse-Geisser and Bonferroni
Chang et al. (2012)	18 CI candidates	0	5.9 (NM)	Profound	Prelingual	NM	NM	3	DTI	ROI	MNI	Uncorrected
Emmorey et al. (2003)	25	25	28.3 (4.5)	Moderate to severe	Congenital	HA > 2	NSL	1.5	VA	ROI	NM	Uncorrected
Hribar et al. (2014)	14	14	35.4 (6)	Profound	Prelingual	No HA	SL	3	DTI, VBM, SBA, Manual volumetry	Whole brain	Talairach	Uncorrected
Huang et al. (2015)	24 CI candidates	20	4.7 (1.0)	Profound	Prelingual	NM	NM	1.5	DTI	ROI	NM	Uncorrected
Kara et al. (2006)	18	18	41.2 (7.5)	Profound	Prelingual	NM	NM	1.5	CT	ROI	NM	Uncorrected
Karns et al. (2017)	23	26	28 (1.4)	Profound	Congenital	NM	NSL	3	DTI	ROI	NM	Uncorrected
Kim et al. (2009)	13	29	29.3 (6.8)	Profound	Prelingual	HA	NM	3	DTI, VBM	Whole brain	MNI	Corrected (DTI) and uncorrected (VBM)
Kim et al. (2014)	8	11	50.4 (6.1)	Severe to profound	Prelingual	No HA	SL	3	VBM	Whole brain and ROI	MNI	Bonferroni and FDR
Kim et al. (2014)	11	11	50.9 (12.2)	Severe to profound	Postlingual	HA	SpL	3	VBM	Whole brain and ROI	MNI	Bonferroni and FDR
Leporé et al. (2010)	14	1	29.5 (NM)	Profound	Prelingual	NM	NSL	1.5	TBM	Whole brain and ROI	Talairach	Corrected
Li J. et al. (2012)	16	16	14.56 (2.10)	Profound	Prelingual	NM	SL	3	CT, VBM	Whole brain	MNI	FDR
Li et al. (2013)	16	16	14.56 (2.10)	Profound	Prelingual	HA	SL	3	CT, VBM	Whole brain	MNI	FDR
Li et al. (2015)	16	16	14.56 (2.10)	Profound	Prelingual	HA	SL	3	VA	ROI	MNI	Bonferroni
Li Y. et al. (2012)	60	38	21.1 (2.26)	Profound	Congenital	No HA	NSL	3	DTI	Whole brain	MNI	FDR
Li Y. et al. (2012)	36	38	21.5 (1.54)	Profound	Prelingual	No HA	SL	3	DTI	Whole brain	MNI	FDR
Lyness et al. (2014)	13	13 NHSL	39.08 (11.08)	Severe to profound	Congenital	NM	SpL and LSL	1.5	DTI	ROI	NM	FDR
Meyer et al. (2007)	6	6	23.5 (NM)	Profound	Congenital	NM	SL	3	VBM	Whole brain	MNI	Uncorrected
Miao et al. (2013)	16	16	14.56 (2.10)	Profound	Prelingual	HA	SL	3	DTI	Whole brain	MNI	Corrected
Olulade et al. (2014)	15	15	23.4 (3.3)	Profound	Congenital	NM	NSL	3	VBM	Whole brain	MNI	Uncorrected
Olulade et al. (2014)	15	15	28.2 (3.8)	Profound	Congenital	NM	SpL	3	VBM	Whole brain	MNI	Uncorrected
Penhune et al. (2003)	12	10	29 (NM)	Profound	Congenital	NM	NSL	1.5	VBM, VA	Whole brain& ROI	Talairach	Uncorrected
Pénicaud et al. (2013)	23	43	39.2 (12.2)	Severe to profound	Congenital	NM	NSL (9) SL (8) LSL (6)	1.5	VBM	Whole brain and ROI	MNI	Corrected
Shibata (2007)	53	51	21 (NM)	Profound	Prelingual	NM	SL	1.5	VBM	Whole brain	MNI	Bonferroni
Smith et al. (2011)	16 CI candidates	26	1.167 (0.25)	Moderate to severe	Congenital	NM	NM	3	VBM, MVA	Whole brain and ROI	MNI	FDR
Smittenaar et al. (2016)	14	15 NHSL	39 (10.2)	Severe to profound	Congenital	NM	LSL	1.5	CT	Whole brain and ROI	NM	Greenhouse-Geisser
Wu et al. (2016)	92 CI candidates	0	4.9 (NM)	Profound	Prelingual	NM	NM	1.5	DTI	ROI	NM	Uncorrected
Zheng et al. (2017)	72/20 CI candidates	38 NH	4.7 (1.0)	Severe to profound	Prelingual	HA	NM	3	DKI	ROI	NM	Uncorrected

*CI, Cochlear implant candidate; CODA, Children of deaf adults; NH, Normo-hearing; NHSL, Normo-hearing sign language users; NSL, Native sign language; SL, Sign language; LSL, Late acquired sign language; SpL, Spoken Language; VBM, Voxel Based Morphometry; TBM, Tensor Based Morphometry; CT, Cortical Thickness; DTI, Diffusion tensor imaging; DKI, Diffusion kurtosis imaging; SBA, Surface based analysis; VA, Volumetric analysis; MVA, Morphometric/Volumetric analysis; ROI, Region of interest; MNI, Montreal Neurological Institute; FDR, False discovery rate; NM, Not mentioned*.

## Results

A total of 1,273 articles were identified from the databases by using the selected keywords. Once the duplicates were removed, 764 articles were included in the selection process. After screening, 27 studies were eligible. Figure 1 shows the selection process according to the guidelines established by PRISMA (Moher et al., 2009).

### Study Selection and Participant Characteristics

The 27 studies were published between the years 2003 and 2017. Five studies reported data acquired using a combination of neuroimaging techniques. Nine studies used morphometric and volumetric analyses, and four studies referenced CT. VBM findings were described in 10 studies, whereas data acquired using DTI were reported in eight studies. Finally, a single study reported data obtained via TBM and one study via DKI. The MRI scanners had a magnetic field intensity of 1.5 Tesla for 12 studies and 3 Tesla for 15 studies. Analysis procedures differed from one study to another and also depended on the neuroimaging technique. Thus, ROI analyses were conducted in 15 studies, whereas whole brain analyses were reported in 12 studies.

The compilation of study data showed acquisition of MRI data in 626 individuals presenting moderate-to-profound bilateral deafness, including 254 children and adolescents. Regarding deafness type, 14 studies focused on congenitally deaf individuals, 13 studies were conducted with pre-lingual deaf individuals, and one study reported data from deaf individuals with post-lingual deafness. With respect to the degree of hearing loss, 20 studies analyzed individuals presenting profound deafness. The degree of hearing loss was considered to be severe to profound in four studies and moderate to profound in three studies. Among the 27 studies, 10 included data from deaf individuals who were native signers, three studies focused on individuals who acquired sign language later in life, and eight studies reported a preferential use of sign language without specifying the time of acquisition. Six studies did not report information regarding the means of communication of participants, although four of them dealt with deaf children who were candidates for cochlear implantation.

Among the 27 selected studies, the majority did not include information regarding the use of hearing aids. Five studies reported absence of hearing aids in the first years of life (Emmorey et al., 2003; Allen et al., 2008, 2013; Kim et al., 2009; Li Y. et al., 2012), whereas two studies noted an absence of hearing aids at the time of testing (Hribar et al., 2014; Amaral et al., 2016). Additionally, five studies reported the use of hearing aids for all participants without indicating the duration of use (Kim et al., 2009; Li et al., 2013, 2015; Miao et al., 2013; Zheng et al., 2017). Finally, four studies presented data of moderate-to-profound deaf children who were scanned while they were candidates for cochlear implant (Chang et al., 2012; Huang et al., 2015; Wu et al., 2016; Zheng et al., 2017).

Most participants in the control groups were hearing individuals. However, three studies presented findings from hearing participants whose primary language was sign language (Allen et al., 2008, 2013; Olulade et al., 2014; hearing individuals born of deaf signer parents). This comparison allows for the measurement of the potential effect of sign language acquired as the first language since birth on brain plasticity by controlling for the impact of auditory deprivation. Two studies reported data of hearing adults who learned sign language (e.g., sign language interpreters).

### Synthesized Findings

**Figure 2:**
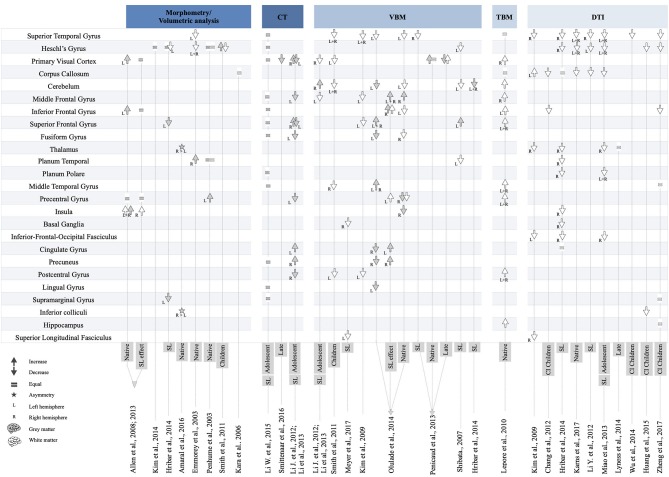
Overview of brain changes in 27 studies on deaf individuals.

To summarize data included in this systematic review, the majority of findings have been categorized according to neuroimaging technique and brain region. In Figure 2, the data are presented in descending order according to the degree of consensus regarding the brain changes reported across the studies. In the next sections, we summarize brain changes according to major brain function and sensory modality.

#### Findings Related to Structures Involved in Auditory and Language Processing

The *superior temporal gyrus* is the brain area most commonly associated with structural modifications in deaf individuals. The superior temporal gyrus is mainly involved in auditory processing, but its left posterior part is specialized in language comprehension (Friederici and Gierhan, 2013). In the right hemisphere, the superior temporal gyrus is implicated in prosodic aspects of speech (Friederici, 2011). Reflected by several neuroimaging techniques, strong evidence supports the presence of white matter changes (reduced volume/density or a reduction in FA values) in the superior temporal gyrus of deaf individuals (Emmorey et al., 2003; Shibata, 2007; Kim et al., 2009, 2014; Smith et al., 2011; Li Y. et al., 2012; Miao et al., 2013; Pénicaud et al., 2013; Hribar et al., 2014; Olulade et al., 2014; Huang et al., 2015; Wu et al., 2016; Karns et al., 2017; Zheng et al., 2017). These changes are found in both hemispheres. Regarding DTI indexes, a reduction in FA appears to be related to an increase in RD in deaf individuals compared with hearing peers (Li Y. et al., 2012; Miao et al., 2013; Wu et al., 2016; Karns et al., 2017). One study reported equal RD and increased AD (Hribar et al., 2014). There is no agreement regarding the gray matter (Li et al., 2015).

The vast majority of reviewed studies report reduced volume, density, and FA in fibers projecting to the primary auditory cortex (*Heschl's gyrus*), which is related to processing speech sounds. These changes are found in the left and right hemispheres (Emmorey et al., 2003; Smith et al., 2011; Li Y. et al., 2012; Miao et al., 2013; Hribar et al., 2014; Huang et al., 2015; Wu et al., 2016; Karns et al., 2017; Zheng et al., 2017). Two studies reported similar white matter volumes between native deaf and hearing individuals (Penhune et al., 2003; Leporé et al., 2010). With regard to DTI, a reduction in FA was found to be related to an increase in RD (Li Y. et al., 2012; Miao et al., 2013; Karns et al., 2017). One study reported no significant difference in RD and AD between deaf and hearing individuals (Hribar et al., 2014). Changes in the gray matter in Heschl's gyrus were also found using morphometry and VBM techniques. One study reported an increase in gray matter density (Smith et al., 2011), whereas another found a decrease (Olulade et al., 2014). Finally, four studies reported similar gray matter volumes of Heschl's gyrus in deaf and hearing individuals (Emmorey et al., 2003; Hribar et al., 2014; Kim et al., 2014; Li et al., 2015).

Although there is an agreement between studies regarding the effects of deafness on the white matter alterations in the superior temporal gyrus and primary auditory cortex, more heterogeneous findings have been reported for other structures. In particular, two types of structural changes have been reported in the inferior frontal gyrus, which is involved in speech production and semantic processing (Friederici and Gierhan, 2013). First, in the left hemisphere, a TBM study reported increased white matter volume in deaf adult native signers (Leporé et al., 2010), whereas three studies observed the opposite effect (Kim et al., 2009; Olulade et al., 2014; Zheng et al., 2017). For the gray matter, a morphometry study reported increased volume in native deaf signers (Allen et al., 2013), whereas other studies reported similar gray matter volume between adolescent signers and hearing individuals (Li et al., 2015).

The *planum temporale*, located above the superior temporal gyrus and partially coinciding with Wernicke's area, is considered to be part of the secondary auditory cortex. For the white matter, two studies reported reduced density and FA in deaf adult signers (Shibata, 2007; Hribar et al., 2014). Alterations in FA were found to be related to a decrease in AD (Hribar et al., 2014). A morphometric study reported similar volumes between native deaf signers and hearing individuals in both the white and gray matter (Penhune et al., 2003), whereas another found an increase in gray matter volume in native adult deaf signers (Emmorey et al., 2003).

Two studies regarding the *planum polare*, which is associated with auditory processing of voice and pitch attributes, reported FA and AD reductions in adolescent and adult signers (Hribar et al., 2014), whereas increases in RD were found (Miao et al., 2013; Hribar et al., 2014).

The *middle temporal gyrus* is involved in linguistic processing and more specifically in lexical–semantic processing (Friederici and Gierhan, 2013). It is also known to be a multimodal area that integrates auditory and visual information (Zatorre, 2002). In this region, two studies reported reduced white matter density in deaf children and native deaf signers (Smith et al., 2011; Olulade et al., 2014), whereas another found no volume differences between deaf and hearing participants (Zheng et al., 2017). One TBM study reported a bilateral increase in white matter volume in native deaf signers (Leporé et al., 2010).

The primary role of the *inferior fronto-occipital fasciculus* is language processing and, more specifically, semantic processing. It mainly connects areas such as the superior and middle frontal cortices, the inferior frontal, and orbitofrontal cortices, and also the superior parietal, angular, and fusiform gyri as well as the occipital lobe. Two studies reported reductions in FA in the right hemisphere of deaf signers (Hribar et al., 2014) and deaf adolescents (Miao et al., 2013), whereas one study found reduced FA in the left hemisphere of prelingually deaf adults (Kim et al., 2009). Hribar et al. (2014) also reported that the reduction in FA is related to a decrease in AD.

The *superior longitudinal fasciculus* connects the frontal and opercular areas with the superior parietal lobe and the angular, supramarginal, and superior temporal gyri. Two studies reported changes in this fasciculus. A reduction in FA in fibers projecting to the right hemisphere was reported in prelingually deaf adults (Kim et al., 2009), whereas a decrease in the white matter in the left hemisphere was shown in congenitally deaf signers (Meyer et al., 2007).

Four studies reported findings in the subcortical auditory pathway that connects the inferior colliculi and medial geniculate nucleus to the auditory cortex. In particular, some studies reported a reduction of FA in fibers projecting to the auditory radiation of deaf children (Chang et al., 2012; Huang et al., 2015), including deaf children under 3 years of age (Zheng et al., 2017). Reduced integrity of the FA was reported in the inferior colliculi of deaf children (Huang et al., 2015), and a rightward asymmetry was found in native deaf signers (Amaral et al., 2016). In the superior olivary nucleus, a reduction in FA was reported in one study (Huang et al., 2015). In deaf individuals, no asymmetry or difference in terms of FA compared with that in hearing individuals was found in the medial geniculate nucleus (Amaral et al., 2016; Zheng et al., 2017), whereas one study found a reduction in FA (Huang et al., 2015).

#### Findings Related to Structures Involved in Visual Processing

The *primary visual cortex* is involved in visual processing, categorization, and various changes have been reported in this region. Thus, three VBM studies reported reduced white matter volume in children and deaf adolescent (Li J. et al., 2012; Li et al., 2013). One TBM study also reported increased white matter volume in native deaf signers (Leporé et al., 2010). Gray matter differences between deaf and hearing individuals have been consistently described in morphometric, CT, and VBM studies. Five reported increased gray matter in this area (Allen et al., 2008, 2013; Li J. et al., 2012; Li et al., 2013; Pénicaud et al., 2013). These findings are observed in the left hemisphere in two studies with native deaf signers (Allen et al., 2013; Pénicaud et al., 2013) and in the right hemisphere in deaf adolescents using sign language (Li et al., 2013). The increase in gray matter volume seems to be related to the use of sign language, as one study reports a reduction in gray matter volume in adult deaf signers who acquired sign language later in life (Pénicaud et al., 2013).

The *fusiform gyrus* is known to play a role in facial recognition and also in recognition of written words. In this area, a single VBM study reported white matter density changes in the right hemisphere of native deaf signers (Olulade et al., 2014). With regard to the gray matter, the findings have been inconsistent. Whereas one study reported similar gray matter volume between adolescent signers and hearing individuals (Li et al., 2015), CT (Li J. et al., 2012; Li et al., 2013) and VBM data suggest a reduction in gray matter density in the left hemisphere of deaf adolescent and adult signers (Olulade et al., 2014).

In the left *lingual gyrus*, Olulade et al. (2014) reported a gray matter density reduction in deaf and hearing adult signers. A subsequent study reported no difference in terms of gray matter density in deaf adolescent signers. A decrease in gray matter volume in the left supramarginal gyrus was also observed in adult deaf signers, whereas Li et al. (2015) found no difference. Finally, one study using DKI reported similar FA in this area between prelingually deaf and hearing children.

#### Findings Related to Structures Involved in Multisensory Processing

The *corpus callosum* is the largest white matter pathway in the brain. Its primary function is to coordinate and allow the interhemispheric transfer of sensory and motor information (Schulte and Müller-Oehring, 2010). In deaf individuals, studies suggest FA reductions of the fibers projecting through the splenium. This reduction was observed in native deaf signers (Karns et al., 2017), in both adolescent and adult sign language users (Li J. et al., 2012; Miao et al., 2013). Two studies reported similar white matter FA in the corpus callosum of deaf individuals and hearing participants, but also reported a decrease in AD (Li Y. et al., 2012; Miao et al., 2013). One study reported increased FA in deaf individuals, located bilaterally in the major forceps of the corpus callosum, which is involved in the interhemispheric transfer of visual information (Kim et al., 2009). Finally, two studies reported reduced FA and increased RD (Miao et al., 2013; Karns et al., 2017), whereas another found reduced AD (Hribar et al., 2014).

The *cerebellum* is known for its role in motor function, as well as posture and balance. It is also involved in cognitive functions such as working memory, long-term memory, implicit and explicit learning, and language (for a review, see Desmond and Fiez, 1998) and has been suggested to be involved in auditory processing (e.g., Petacchi et al., 2005). Three VBM studies reported a decrease in the cerebellum white matter in deaf children and in adults using sign language (Shibata, 2007; Smith et al., 2011; Olulade et al., 2014). One TBM study reported an increase in white matter volume in native deaf signers (Leporé et al., 2010). For the gray matter, two VBM studies reported reduced density in deaf signers and native deaf signers compared with native hearing signers (Hribar et al., 2014; Olulade et al., 2014), although an opposite effect was shown in a study conducted with adolescent signers (Li et al., 2013).

The *thalamus* plays a significant role in the relay and integration of sensory afferences and motor efferences. Three DTI studies reported a reduction of FA in fibers projecting to the right internal capsule next to the thalamus in deaf adults, deaf adolescents, and adult signers. One study also reported reduced AD (Hribar et al., 2014), whereas another reported increases in MD and RD in the frontal and occipital thalamic radiations in late deaf signers (Lyness et al., 2014). A single study reported a rightward volume asymmetry in the thalamus of native deaf signers (Amaral et al., 2016).

The *insula* contributes to several cognitive processes as well as multisensory integration (e.g., Naghavi et al., 2007). One study reported increased gray matter in deaf native signers. Olulade et al. (2014) contradicted this finding by reporting the opposite pattern. As for the white matter, one study reported reduced FA and AD in deaf signers (Hribar et al., 2014). Finally, a leftward asymmetry in the gray matter was reported in native deaf signers compared with native hearing signers in the posterior lobule (Allen et al., 2008).

#### Findings Related to Structures Involved in Motor Processing

Neuroimaging data regarding the *precentral gyrus*, or primary motor cortex, are inconsistent. Whereas, Leporé et al. (2010) reported a bilateral increase in primary motor cortex volume in native deaf signers, another study found increased white matter density in the left hemisphere of native signers (hearing and deaf) (Olulade et al., 2014). A leftward asymmetry was also reported in the hand region in deaf signers, whereas it is typically observed in the right hemisphere of hearing individuals (Allen et al., 2013).

The *basal ganglia* play a role in involuntary motor activity and muscle tone. In these nuclei, two studies (VBM and DTI) reported reduced white matter density in the right hemisphere of deaf adults exclusively using sign language (Meyer et al., 2007; Hribar et al., 2014). A single study reported a gray matter increase in the caudate nucleus of native deaf signers (Olulade et al., 2014).

#### Findings Related to Structures Involved in Higher Cognitive Functions

The *middle frontal gyrus* is associated with higher cognitive functions such as executive functions, memory, and language. Two studies using VBM and CT reported altered white matter density and thickness in the left hemisphere of adolescent signers (Li Y. et al., 2012) and deaf adults (Kim et al., 2009). One TBM study reported an increase of white matter volume in the right hemisphere in native deaf signers (Leporé et al., 2010). One VBM study also found increased gray matter density in the right hemisphere of native deaf signers and native hearing signers, suggesting an effect of sign language in the prefrontal cortex (Olulade et al., 2014).

The *superior frontal gyrus* is primarily involved in higher cognitive functions and, more specifically, in working memory (du Boisgueheneuc et al., 2006). Regarding the white matter, findings are inconsistent. One VBM study reported a leftward decrease of white matter density in deaf adults (Kim et al., 2009). One TBM study also found a bilateral increase in white matter volume in the superior frontal gyrus in native deaf signers (Leporé et al., 2010). With regard to gray matter, two VBM studies and one CT study reported gray matter increases in the right hemisphere of deaf signers (Shibata, 2007; Li Y. et al., 2012; Li et al., 2013; Olulade et al., 2014). This effect seems to be associated with sign language, as it is also observed in native hearing signers. Finally, three morphometric studies reported opposite results, with gray matter reductions in the superior frontal gyrus of deaf signers (Li J. et al., 2012; Li et al., 2013; Hribar et al., 2014).

#### Findings From Other Structures Without Consistent Observations

In the *postcentral gyrus* or primary somatosensory cortex, two studies reported reduced white matter in deaf children (Smith et al., 2011) and adults (Kim et al., 2009), whereas one TBM study reported a bilateral increase in white matter volume in native deaf signers (Leporé et al., 2010). For the gray matter, a single study found reduced CT in deaf adolescent signers (Li J. et al., 2012).

In the *cingulate gyrus*, inconsistent findings were reported. One study found increased gray matter volume in the left hemisphere of deaf adolescent signers and a decrease of gray matter density in the right hemisphere of native deaf and hearing signers (Olulade et al., 2014). One DTI study found similar FA and RD and a bilateral reduction in AD in the anterior region of the cingulate gyrus (Hribar et al., 2014).

In the *precuneus*, an increase in gray matter volume was reported in the right hemisphere of deaf signers (Olulade et al., 2014). Another study reported similar gray matter volume between deaf adolescent signers and hearing individuals (Li et al., 2015).

## Discussion

The aim of the present systematic review was to identify key features of structural plasticity in deaf individuals by examining cerebral changes in the gray and white matter. We provide an up-to-date synthesis with a focus on structural changes identified with the following neuroimaging techniques: volumetry, VBM, TBM, CT, DTI, and DKI. With the use of the PRISMA method (Moher et al., 2009), 27 papers were selected that describe the structural changes reported in 626 individuals with a moderate-to-profound bilateral deafness, including 254 children and adolescents. This review provides converging evidence from several studies to determine specific or consistent changes in the gray and white matter in congenital and prelingual deaf individuals. As the plasticity of the gray and white matter is experience dependent, the ontogenetic events occurring throughout development must be considered in the context of sensory loss. We thus emphasize the effect of auditory deprivation and more specifically the consequences of the early absence of aural experiences on the long-term development of brain anatomy.

### Summary of Main Findings

#### Cerebral Changes Induced by Auditory Deprivation

Nearly all studies included in this review focused on cortical regions implicated in auditory processing: the primary auditory cortex (Heschl's gyrus) and secondary auditory cortex (planum polare and planum temporale). Evidence from these studies shows white matter changes across all these areas. Specifically, reduced white matter volume and density, as well as reduced FA, were observed in deaf children, adolescents, and adults. For the superior temporal gyrus, which is involved in language processing, the majority of studies reported bilateral white matter changes in volume, density, and FA. A large body of work suggests that the early absence of auditory stimulation leads to reduced myelinization in these areas (e.g., Hribar et al., 2014; Karns et al., 2017). Additionally, findings suggest that these changes are not sensitive to the means of communication used by deaf individuals (i.e., spoken or sign language). However, they are negatively correlated with auditory and speech perception in children who were candidate for cochlear implantation. Indeed, those with the poorest perceptive abilities after implantation show greater and broader changes in the primary and secondary auditory areas.

Additional cortical and subcortical structures, rarely discussed in the context of neuroplasticity in deaf individuals, also contribute to auditory processing and are modified by auditory deprivation. Hence, white matter changes have been reported in the posterior part of the corpus callosum (or splenium), which allows interhemispheric connections between auditory areas (Zatorre, 2002). Anatomical differences have also been described in the anterior portion of the corpus callosum (or genu), which connects the left and right prefrontal and orbitofrontal regions (Chang et al., 2012). These changes are negatively correlated with the auditory perceptive abilities of children candidates for cochlear implantation. One study showed a bilateral increase in the white matter in the splenium, a portion of the corpus callosum involved in interhemispheric visual association (Kim et al., 2009). Changes in subcortical structures implicated in the auditory functions were also found. Reduced FA was observed in fibers projecting to the auditory radiation, the superior olivary nucleus, and the inferior colliculi. All of these changes are correlated with the speech perception outcomes of children fitted with a cochlear implant. With regard to the gray matter, a rightward volume asymmetry in native deaf signers was reported in subcortical structures (inferior colliculi and thalamus; Amaral et al., 2016). These asymmetries are interpreted as constituting mechanism for the transmission of visual information toward the auditory regions in deaf individuals (Amaral et al., 2016).

In sum, the evidence demonstrates significant changes in the main cortical and subcortical structures implicated in auditory processing, which appear to be present from an early age and have long-lasting effects. However, Li Y. et al. (2012) reported a significant correlation between FA in fibers projecting to the superior temporal gyrus and the age of deafness onset. This result is consistent with the presence of a critical developmental period that is sensitive to auditory deprivation during postnatal life and critical for rehabilitation strategies (Kral, 2013). As it relates to experience-dependent plasticity, one open question is whether the use of hearing aids modifies the extent of the reported structural changes. This question deserves to be thoroughly investigated because a relationship between the duration of hearing aid use and the extent of functional reorganization in the auditory cortex has only been shown in a functional connectivity study (Shiell et al., 2015).

### Cerebral Changes Related to Language

Numerous brain areas and circuits are involved in language processing and production in the human brain (Friederici, 2011). Among these, the inferior frontal cortex, the superior temporal gyrus, and the middle temporal gyrus are believed to be the most important (Friederici, 2011). Typically, language production also requires the contribution of premotor and motor regions, whereas language perception implicates the auditory and visual systems. The present review confirms the presence of brain changes in language-related areas in deaf individuals. There was strong evidence for bilateral white matter changes in volume, density, and FA in the superior temporal gyrus. Reductions in white matter volume and FA in the inferior frontal gyrus of the left hemisphere were also reported. However, the data do not provide robust evidence of middle temporal gyrus structural changes.

Four major fasciculi are involved in language processing. The dorsal pathway, connecting the frontal and temporal regions, includes the arcuate fasciculus and parts of the superior longitudinal fasciculus. These two fasciculi are involved in syntactic and speech repetition (Dick et al., 2014). The ventral pathway includes the uncinate fasciculus, also implicated in the primary syntactic process, and the inferior fronto-occipital fasciculus, involved in semantic and comprehension processing (Dick et al., 2014). In deaf individuals, only some studies reported reduced density of FA in the superior longitudinal fasciculus, the uncinate fasciculus and the inferior fronto-occipital fasciculus; and none presented findings regarding fibers projecting in the arcuate fasciculus. This demonstrates the need for future studies to evaluate the role of the ventral and dorsal language pathways in deaf individuals.

Moreover, three additional structures involved in language processing could be impacted by sensory deprivation and should be the focus of further research. Indeed, the supramarginal gyrus, which contributes to the processing of prosody, could present white and gray matter abnormalities. The angular gyrus, which is involved in semantic processing, word reading, and comprehension, has been shown to be altered in a single study with children who were candidate for cochlear implantation (Zheng et al., 2017). Beyond these language functions, the angular gyrus is an essential structure in the context of sensory deprivation, as it is a cross-modal hub where sensory information (auditory, visual, and tactile) converges and is integrated (Seghier, 2013). The insula is an important structure involved in auditory processing and the motor aspects of speech. More importantly, this structure also plays a role in multisensory integration at the level of audio-visual and visuo-tactile integration (e.g., Naghavi et al., 2007). Inconsistent findings are reported across the studies included in this review for both the gray and white matter, although auditory deprivation seems to increase the gray matter in the posterior insula. According to Allen et al. (2008), this change could be related to increased use of visual speech reading or stronger articulatory-based phonological representations of speech.

The reviewed data suggest that it may be necessary to differentiate structural changes according to means of communication (spoken or sign language). At the cerebral level, sign and spoken language share common neural bases, although some specificities have been reported. For example, higher activation of the posterior middle temporal gyri is observed in sign language when compared with spoken language (MacSweeney et al., 2008). The majority of studies detailed in this review involved deaf individuals who preferentially use sign language. However, in 10 studies, deaf participants were born of deaf parents and used sign language exclusively as a means of communication. These deaf signers only represent 5% of the total deaf population, and their linguistic abilities cannot be related to the majority of deaf individuals born in hearing families (Bavelier et al., 2006). Here, sign language appears to be a confounding factor when extrapolating functional information from anatomical changes. Nevertheless, comparing deaf individuals with a group of hearing native signers can isolate the effect of sign language that interacts with the effect of auditory deprivation. The present review identified three studies (Allen et al., 2008, 2013; Olulade et al., 2014) that directly compared the brain anatomy of deaf native signers with hearing native signers. Early acquisition of sign language is associated with increased volume or density of the gray and white matter in regions implicated in language processing (inferior frontal gyrus), executive functions (middle frontal gyrus), visuospatial and motor processing (precuneus and precentral gyrus), and multimodal sensory integration (insula). Deaf native signers also present specific brain differences in regions involved in auditory and language processing (superior temporal gyrus, inferior frontal gyrus, and middle temporal gyrus) and executive functions (middle frontal gyrus), and also in visual (fusiform gyrus and calcarine sulcus), motor/sensorimotor (precentral gyrus, cerebellum, and caudate), and multisensory integrative areas (insula).

These findings suggest that early auditory deprivation leads to specific brain changes according to the means of communication (spoken or sign language). In young deaf children who are candidates for cochlear implantation, lower auditory perception scores are correlated with a decrease in FA in regions involved in linguistic processing (superior temporal gyrus, Heschl's gyrus, angular gyrus, genu of corpus callosum, and inferior frontal gyrus). These changes support the auditory deprivation hypothesis, which suggests that the absence or deterioration of auditory experience impacts the development of speech and spoken language as well as other cognitive functions such as executive functions (Beer et al., 2011). A second hypothesis suggests that this neurodevelopmental cascade can be explained by early language deprivation. This situation is often seen in deaf individuals for whom the auditory deficiency is only detected once the acquisition of spoken language abilities is visibly delayed and is also often associated to altered executive functioning (e.g., Figueras et al., 2008; Kral et al., 2016). A recent study has shown that native deaf signer children have similar executive functioning as hearing children matched by age and gender (Hall et al., 2017). Therefore, learning sign language appears to be associated with specific structural plasticity in multiple brain areas. This could act as a protection factor, minimizing the effect of auditory deprivation on neurocognitive development.

### Cerebral Changes Induced by Compensatory Mechanisms

When comparing deaf and hearing individuals, numerous studies have reported enhanced abilities in deaf individuals in various sensory tasks, such as visual ones (Levänen and Hamdorf, 2001; e.g., Dye et al., 2007; Shiell et al., 2014a); higher cognitive functions, such as attention orientation (Colmenero et al., 2004); and recognition of emotional expressions and facial features (Bettger et al., 1997; Arnold and Murray, 1998). The principal explanation is that these behavioral enhancements are supported by cross-modal activations of auditory regions (Merabet and Pascual-Leone, 2009). Cross-modal plasticity refers to the recruitment of affected cortical areas by another sensory modality (Kral et al., 2019). The review of previously published observations explains certain sensory compensatory mechanisms with structural plasticity in individuals with early auditory deprivation. Studies reviewed here suggest that gray matter changes may be associated with visual experience in deaf individuals. In fact, several functional neuroimaging and behavioral studies suggest that congenitally or early deaf individuals possess enhanced abilities for visual localization (for a review, see Pavani and Bottari, 2012) and visual motion detection (Shiell et al., 2014a). The present review supports the general agreement in VBM and volumetric studies in terms of gray matter increases in the visual areas of native deaf signers, leading to enhanced visual abilities (Allen et al., 2013; Pénicaud et al., 2013). It has also been suggested that an increase in the gray matter is an effect not only of auditory deprivation but also of early sign language exposure, as demonstrated by contrasting native deaf signers with late deaf signers. Indeed, one study reported gray matter reductions in the primary visual cortex of late deaf signers (Pénicaud et al., 2013). By apparent contrast, one study reported increased visual performance in the peripheral visual field, which was associated with thickness reduction in the primary visual cortex of deaf individuals (Smittenaar et al., 2016).

Atypical somatosensory change has also been observed when comparing deaf and hearing individuals, where deafness-induced cross-modal plasticity seems to support enhanced performance (e.g., Levänen and Hamdorf, 2001; Heimler and Pavani, 2014). Regarding motor development, a single study reported a delay in fine motor skill development in prelingually deaf children (Horn et al., 2006). However, in regions involved in motor and somatosensory processing, there is currently no consensus as to gray or white matter changes. Also, when looking at the post-central gyrus or primary somatosensory cortex, a single study identified reduced gray matter density in deaf adolescents (Li J. et al., 2012).

To explore the relationship between functional and structural reorganization induced by auditory deprivation, it appears necessary to develop protocols that include specific and sensitive behavioral tasks associated with their anatomical neural substrates. For example, one recent study reported that an increase in CT in the right posterior superior temporal cortex was associated with visual motion detection abilities in early and profoundly deaf individuals (Shiell and Zatorre, 2016). For their part, Smittenaar et al. (2016) reported enhanced peripheral vision in congenitally deaf adults associated with reduced CT in the primary visual cortex.

### Issues Regarding the Interpretation of Brain Plasticity Data

When discussing cortical reorganization, certain general aspects of cerebral plasticity could help the interpretation of the reported results. Indeed, recently, several studies have identified cortical changes as a result of experience-dependent plasticity. For example, when considering the gray matter volume changes in individuals learning a musical skill before and after extensive practice, it is reported that the musical experience affects regions involved in higher cognitive processes such as executive functions, memory, or emotions (e.g., Groussard et al., 2014). Accordingly, an increase in the gray matter in somatosensory and auditory areas is usually interpreted as an adaptive plasticity phenomenon leading to enhanced performances, as demonstrated in opera singers (Kleber et al., 2016). However, a study shows contradictory results with a rapid increase in gray matter density in sensorimotor-related brain areas followed by a decrease after a few training sessions with a complex whole-body balancing task (Taubert et al., 2010). A careful interpretation is thus necessary regarding brain–behavior relationships when looking at gray matter differences because contradictory findings lead to the hypothesis that plasticity is functionally selective (Heimler et al., 2014).

Concerning the white matter, the DTI technique is currently a powerful instrument for the study of anatomical correlates and changes at the levels of fibers (diameter and density) or myelinization. However, DTI is a relatively complex neuroimaging technique given the intricate nature of the white matter and the extensive available choices of analyses. This complexity leads to many misconceptions regarding the interpretation of results (for an extensive review, see Jones et al., 2013). A vast number of studies addressing clinical populations show white matter alteration, for example, Alzheimer's disease (Damoiseaux et al., 2009), schizophrenia (Qiu et al., 2010), and Tourette's syndrome (Neuner et al., 2010). In a neurotypical population, extensive piano practice is associated with an increased myelinization in children and is maintained with age (Bengtsson et al., 2005). Most studies in this review only report changes to the FA. The RD, which measures index levels of myelinization, and the AD, which reflects the integrity of microtubules along the axon, seem necessary for an exhaustive understanding of white matter plasticity. For example, in deaf individuals, three studies indicate that superior temporal gyrus reductions in FA following deafness can be better attributed to changes in RD than in AD (Li Y. et al., 2012; Miao et al., 2013; Karns et al., 2017). A single study reports differences in the AD (Hribar et al., 2014). On the other hand, a reduction of FA in regions implicated in auditory and lingual processing appears to be consistent across the studies reported in this systematic review. However, heterogeneity in complementary measures (AD, RD, and MD) suggests the importance of follow-up DTI studies.

Finally, an increase in CT seems to be associated with groups of neurons missing their migrating targets in the cerebral cortex leading to the formation of a neuronal nodule (Guerrini and Marini, 2006). These authors propose a second hypothesis to explain structural abnormalities with the presence of polymicrogyria, an excessive number of convolutions distanced by enlarged sulci (Guerrini and Marini, 2006). An increase in CT is therefore associated with a maladaptive plasticity process and is identified among several neurodevelopmental disorders such as reading impairment (Chang et al., 2005) and congenital amusia (Hyde et al., 2007).

### Limitations

The diversity of developmental deafness profiles, observed in the 27 reviewed studies, considerably restricts generalization of the reported effects to the entire deaf population. Factors such as deafness onset, deafness duration, age of language acquisition, degree of hearing loss, amount of residual hearing, and use of hearing aids should ideally be considered in future analyses. Some of the studies in this review assessed cerebral changes relative to age of language acquisition (sign language) (Miao et al., 2013; Lyness et al., 2014) or age of onset vs. duration of deafness (Li Y. et al., 2012). Evidently, a larger sample size is necessary to adequately consider these multiple variables. Sample size is an important challenge in this area of research, as some of the reviewed studies reported findings from the same group of deaf individuals using different neuroimaging techniques. Also, all of the reviewed studies used a cross-sectional design. Longitudinal studies are needed to better understand the time course of deafness-related structural changes and to reduce the heterogeneity of deafness profiles. Long-term follow-ups would also allow for identification of structural changes as a function of means of communication or help determine optimal rehabilitation strategies.

Multiple constraints also concern the neuroimaging techniques themselves, their limits, and the various types of analyses. Whereas, some studies used a whole-brain approach, others focused on ROIs, based on previous research. In the context of this review, whole-brain interpretations of differences between deaf and hearing individuals were based solely on published findings that often omit to report null findings. Moreover, both methods require different correction approaches (Genovese et al., 2002), and a substantial number of reviewed studies did not apply correction methods to their findings.

## Conclusion

The present systematic review aimed at regrouping the current scientific literature on brain changes following early auditory deprivation from 27 studies on 372 deaf adults and 254 deaf children. Auditory deprivation primarily alters brain structures of the primary and secondary auditory cortex and language areas. These structural changes appear to be modulated by individual variables (deafness onset, deafness duration, and means of communication) and to influence behavioral performance during sensory and cognitive tasks. Many of these changes in cortical and subcortical auditory and language areas are negatively correlated with auditory and speech perception ability in deaf children with a cochlear implant. Therefore, further neuroimaging studies are required to distinguish the heterogeneity in auditory and language outcomes in deaf children with a cochlear implant and, moreover, to optimize clinical prognosis and rehabilitation. Furthermore, early acquisition of sign language appeared to increase the gray and white matter in both deaf and hearing individuals. Consequently, the learning of sign language could be used as a protective factor in the neurocognitive development of deaf children. Nevertheless, the effect of sign language on neurodevelopmental outcomes of deaf children remains open for discussion.

Finally, we argue that some of the inconsistent findings may be related to deafness variables and methodological limitations of the reported neuroimaging studies. Therefore, future studies are needed to establish “best practice” guidelines for the analysis of structural brain changes in deaf individuals. To counter the issue restricting generalization, we suggest well-powered studies and adding a hearing native signers' group to isolate the confounded effects of sign language and auditory deprivation. We also propose longitudinal studies comprising behavioral tasks that could help develop better rehabilitation strategies for deaf individuals.

## Data Availability Statement

The raw data supporting the conclusions of this article will be made available by the authors, without undue reservation, to any qualified researcher.

## Author Contributions

MS and FG performed the studies selection and the data collection and designed figures and tables. MS, FG, and EC drafted the manuscript. FC, MWM, and FL reviewed the manuscript.

### Conflict of Interest

The authors declare that the research was conducted in the absence of any commercial or financial relationships that could be construed as a potential conflict of interest.
